# A Feasibility Study for CODE-MI: High-Sensitivity Cardiac Troponin - Optimizing the Diagnosis of Acute Myocardial Infarction/Injury in Women.

**DOI:** 10.23889/ijpds.v7i3.1815

**Published:** 2022-08-25

**Authors:** Yinshan Zhao, Atul Sivaswamy, May K. Lee, Mona Izadnegahdar, Anna Chu, Laura E. Ferreira-Legere, Karin H. Humphries, Jacob A. Udell

**Affiliations:** 1 Population Data BC; 2 ICES; 3 Centre for Improved Cardiovascular Health at Centre for Health Evaluation and Outcome Sciences; 4 Division of Cardiology, Univeristy of British Columbia; 5 University of Toronto

## Objectives

To contribute to the evidence on the association between neighbourhood-level child development in Kindergarten and neighbourhood SES, our objective was to quantify the sociodemographic and child development characteristics of the neighbourhoods that “defy expectations”: high SES neighbourhoods with much-worse-than-expected child outcomes, and low SES neighbourhoods with much-better-than-expected child outcomes.

## Approach

Using exploratory and model-based Latent Profile Analysis (LPA), we identified homogenous profile groups of 2038 customized Canadian neighbourhoods using ten SES indicators. We identified the most parsimonious number of profile groups and validated and characterized the derived groups of neighbourhoods using neighbourhood and aggregated child characteristics. Next, as our outcome, we created quartile groups for developmental vulnerability risk, measured with the Early Development Instrument (EDI), to match the number of derived neighbourhood profile groups. Last, we used contingency table analysis to identify neighbourhoods that defy expectations, and then characterized these neighbourhoods using descriptive statistics and correlational analysis.

## Results

The LPA identified four neighbourhood SES groups which we labelled “Low” (31.6%), “Low-moderate” (12.7%), “High-moderate” (38.4%) and “High” (17.4%). These four SES groups were cross-tabulated with quartile groups of EDI vulnerability risk. Inspection of the resulting 4-by-4 contingency table showed that within the “Low” SES profile group 57 (8.9%) neighbourhoods had much-better-than-expected developmental vulnerability risk. Conversely, within the “High” SES profile group, 12 (3.4%) neighbourhood had much-worse-than-expected developmental vulnerability risk. Additionally, these analyses identified large provincial differences in the proportion of neighbourhoods that defy expectation. In 12 provinces and territories in the study, the proportion of neighbourhoods that defied expectations within each province ranged from zero to 50%.

## Conclusion

The identification of neighbourhoods that defy expectations contributes to our understanding of neighbourhood factors influencing child development. Using mixed-methods approaches, these neighbourhoods can be compared to nearby neighbourhoods from the same SES profile group that do not defy expectations, in an effort to identify contextual factors that differentiate them.

